# iPiDA-LGE: a local and global graph ensemble learning framework for identifying piRNA-disease associations

**DOI:** 10.1186/s12915-025-02221-y

**Published:** 2025-05-09

**Authors:** Hang Wei, Jialu Hou, Yumeng Liu, Alexey K. Shaytan, Bin Liu, Hao Wu

**Affiliations:** 1 School of Computer Science and Technology, Xidian University, Xi’an, Shaanxi 710126 China; 2 SMBU-MSU-BIT Joint Laboratory On Bioinformatics and Engineering Biology, Shenzhen MSU-BIT University, Shenzhen, Guangdong 518172 China; 3 School of Computer Science and Technology, Beijing Institute of Technology, Beijing, 100081 China; 4 College of Big Data and Internet, Shenzhen Technology University, Shenzhen, Guangdong 518118 China; 5 Department of Biology, Lomonosov Moscow State University, Moscow, 119234 Russia; 6 International Laboratory of Bioinformatics, AI and Digital Sciences Institute, Faculty of Computer Science, HSE University, Moscow, 109028 Russia; 7 Zhongguancun Academy, Beijing, 100094 China

**Keywords:** piRNA-disease association identification, Graph ensemble learning, Local context graph

## Abstract

**Background:**

Exploring piRNA-disease associations can help discover candidate diagnostic or prognostic biomarkers and therapeutic targets. Several computational methods have been presented for identifying associations between piRNAs and diseases. However, the existing methods encounter challenges such as over-smoothing in feature learning and overlooking specific local proximity relationships, resulting in limited representation of piRNA-disease pairs and insufficient detection of association patterns.

**Results:**

In this study, we propose a novel computational method called iPiDA-LGE for piRNA-disease association identification. iPiDA-LGE comprises two graph convolutional neural network modules based on local and global piRNA-disease graphs, aimed at capturing specific and general features of piRNA-disease pairs. Additionally, it integrates their refined and macroscopic inferences to derive the final prediction result.

**Conclusions:**

The experimental results show that iPiDA-LGE effectively leverages the advantages of both local and global graph learning, thereby achieving more discriminative pair representation and superior predictive performance.

**Supplementary Information:**

The online version contains supplementary material available at 10.1186/s12915-025-02221-y.

## Background

PIWI-interacting RNA (piRNA) is a category of small non-coding RNA molecules with high conservation, species specificity, and abundant expression [1]. It is involved in multiple biological functions like transposable element silencing, gene expression regulation, embryonic development, and epigenetic modification to maintain genome stability and reproductive process by forming complexes with members of the PIWI protein family [2–4].

As piRNAs are the critical regulatory factors in biological processes, their function abnormality may promote occurrence and development of diseases [5–8]. Several studies have been conducted to explore piRNA functions [9, 10]. For example, piRNA-eQTL is the first robust eQTL database that systematically uncovers the effects of genetic variants on piRNA expression across various cancer types [11]. PiRSNP identified human and mouse piRNA-related SNPs and evaluated their impacts on piRNA-mRNA binding [7]. PiRTarBase offers predicted and experimentally identified functional piRNA targeting sites [12].

Recent studies have shown that piRNAs can serve as promising diagnostic or prognostic biomarkers and therapeutic targets for various cancers [13–15]. Therefore, numerous computational approaches have been suggested for detecting potential piRNA-disease associations, laying the groundwork for subsequent biological investigations. These approaches can primarily be categorized into two groups: classical machine-learning-based and graph-learning-based approaches [16, 17]. Most classical machine-learning-based methods manually splice the attribute information of piRNAs and diseases to construct pair features. To overcome the instability of a single classifier, iPiDA-PUL identified associations based on an ensemble of various classical classifiers [18], and iPiDA-LTR integrated different feature components through a supervised ranking framework [19]. iPiDA-sHN was proposed to improve the quality of negative pairs based on two-step positive-unlabeled learning [20]. With the successful application of graph neural networks in bioinformatics tasks [21–23], several graph-learning-based methods have been proposed to explore the hidden structural features and association patterns within piRNA-disease association networks. For example, iPiDA-GCN designed two principal sub-modules for iteratively learning the proximity features of homogeneous similarity networks and heterogeneous piRNA-disease bipartite network [24]. Considering the varying importance among bio-entity nodes, ETGPDA and PUTransGCN constructed heterogeneous networks and employed different attention-aware graph neural networks to identify potential piRNA-disease associations [25, 26]. The known piRNA-disease associations are relatively limited, posing a challenge due to the highly sparse nature of the association network. To enhance network structural semantics, iPiDA-SWGCN incorporated a supplemental weighted strategy [27], CLPiDA integrated LightGCN with a data augmentation technique [28], and iSG-PDA fused multi-source genetic information [29].

Despite the significant advancements made by the aforementioned graph-learning-based methods in predicting piRNA-disease associations, several issues still need to be addressed: (i) Current methods integrate proximity information from the global piRNA-disease network during the learning process, resulting in more informative node features. However, it may introduce irrelevant noise interference by considering the entire graph structure in each layer, causing over-smoothing of node features. (ii) Existing methods overlook local proximity relationships, which are crucial for piRNA-disease association identification task. PiRNAs may exhibit diverse functional mechanisms across different diseases [9]. However, global graph learning can only extract general and globally invariant node features, making it difficult to detect discriminative association patterns [30, 31]. Overall, fully exploring latent biological semantics while alleviating noise interference and enhancing the discriminative ability of piRNA-disease pair representation remains a challenge.

To overcome the aforementioned limitations, we propose a new method named iPiDA-LGE for identifying piRNA-disease associations based on local and global graph ensemble learning. iPiDA-LGE designs two primary graph convolutional neural network modules to capture local-specific features and general features, thereby enhancing the expressiveness of piRNA-disease pairs. Subsequently, it integrates prediction results derived from refined and macroscopic inferences based on local and global piRNA-disease association networks, respectively. Experimental results indicate that iPiDA-LGE can simultaneously maintain the advantages of local and global graph learning, facilitating a more precise and comprehensive identification of piRNA-disease associations.

## Results and discussion

### Parameter analysis

iPiDA-LGE comprises two primary modules including global-level graph learning and local-level graph learning. Given the parameters in global-level graph learning have been discussed in our previous study [27], we focus on analyzing parameters in local-level graph learning in this study. The impact of four important parameters including neighbor order, epoch, learning rate, and GCN layer is discussed.

**Fig. 1:**
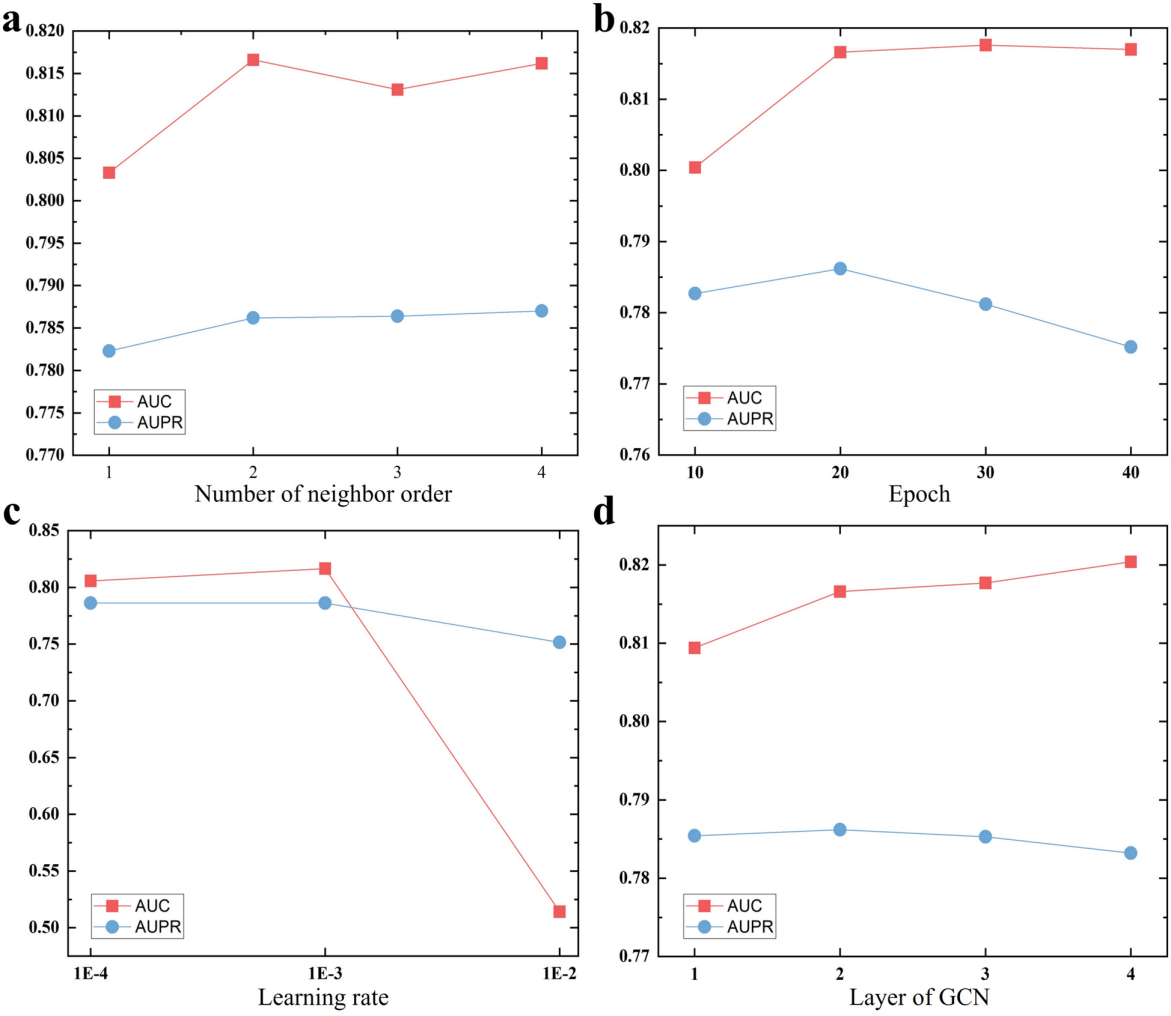
Parameter analysis of local-level graph learning in iPiDA-LGE. **a**, **b**, **c**, and **d** illustrate the AUC and AUPR obtained by local-level graph learning module with different neighbor orders, epochs, learning rates, and GCN layers on ${\mathbb{S}}_{\text{validation}}$, respectively

As shown in Fig. 1, the results reveal the following observations: (i) The number of neighbor order influences the size and contextual scope of each local piRNA-disease graph; there is limited contextual semantic information when selecting 1-hop neighborhood to extract local piRNA-disease graph. (ii) The performance of local-level graph learning module initially improves and then decreases with the increment of epochs attributed to overfitting, and a large learning rate may cause divergence or oscillated around the optimal solution. (iii) Different from global-level graph learning, the local-level graph learning module only captures local topology structure and is relatively less sensitive with GCN layer. Ultimately, the neighbor order, epoch, learning rate, and GCN layer are set to 2, 20, 0.001, and 2 during local-level graph learning with considering runtime and predictive performance.

### Global and local graph ensemble learning can improve the predictive performance

**Fig. 2:**
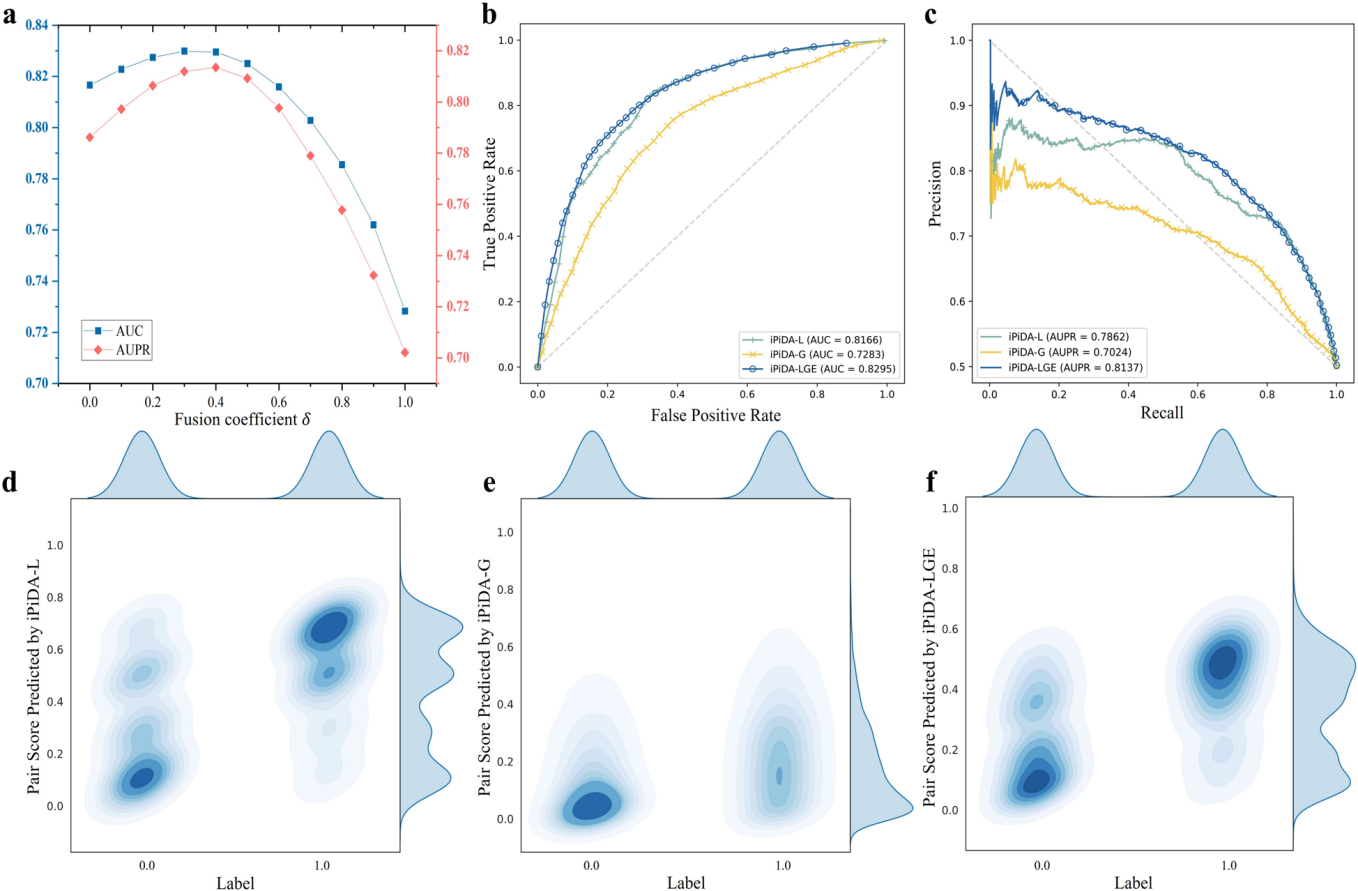
Comparison between local/global graph learning and their combination. **a** shows the AUC and AUPR achieved by iPiDA-LGE with different fusion coefficients on ${\mathbb{S}}_{\text{validation}}$. **b** and **c** show the ROC and PR curves obtained by different predictors on ${\mathbb{S}}_{\text{validation}}$, respectively. **d**–**f** show the binary distribution of true labels and association scores predicted by iPiDA-L, iPiDA-G, and iPiDA-LGE on ${\mathbb{S}}_{\text{validation}}$

To study if ensemble learning of local and global piRNA-disease association graphs could improve the predictive performance, two baseline predictors, iPiDA-L and iPiDA-G, which are learned from local and global graph levels are compared with iPiDA-LGE. The comparison results are depicted in Fig. 2, enabling us to draw the following conclusions: (i) The fusion coefficient is relativity sensitive to the overall performance of iPiDA-LGE. Compared to iPiDA-G, iPiDA-L based on local graph learning plays a more important role in iPiDA-LGE and achieves higher predictive performance, attributing to its ability to capture specific contextual semantics for each target piRNA-disease pair and preventing from noise interference. (ii) iPiDA-LGE is superior to iPiDA-L and iPiDA-G regarding AUC and AUPR metrics, illustrating ensemble learning of local and global piRNA-disease association graphs contributes to performance improvement. (iii) iPiDA-L may predict some false positive associations, while iPiDA-G tends to predict potential piRNA-disease associations as negatives. iPiDA-LGE can obtain more discriminative association scores by integrating iPiDA-L and iPiDA-G.

### Feature analysis of local and global graph representation

There are different types of piRNA-disease pair features including initial attribute features, local and global graph structural features. To analyze their contributions for identifying piRNA-disease associations, four predictors based on various features are constructed and compared. Table 1 lists the performance results obtained by iPiDA-LGE along with iPiDA-A, iPiDA-L, and iPiDA-G on ${\mathbb{S}}_{\text{independent}}$, where iPiDA-A is a random forest-based predictor trained by concatenating piRNA and disease attribute features. It is not surprising that the AUC and AUPR obtained by iPiDA-A is significantly lower than other predictors based on graph structural features. In addition, consistent with above observation on the benchmark dataset, iPiDA-LGE achieves superior performance owing to its local and global graph ensemble learning.

**Table 1:** Performance comparison of iPiDA-LGE with baseline methods on ${\mathbb{S}}_{\text{independent}}$

Method	AUC	AUPR
iPiDA-A	0.6321	0.5838
iPiDA-L	0.8402	0.8231
iPiDA-G	0.8178	0.8151
iPiDA-LGE	0.8537	0.8497

**Fig. 3:**
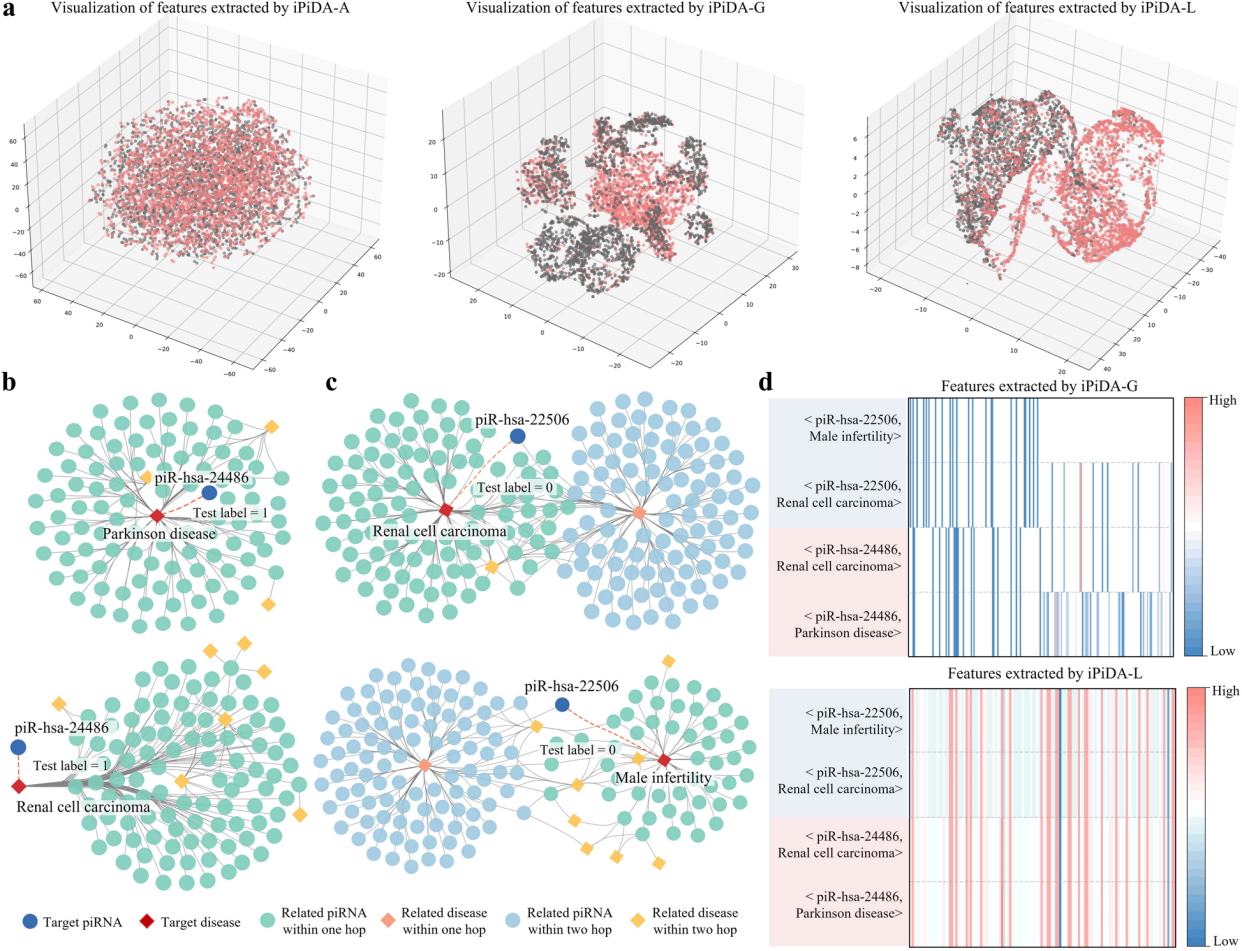
Analysis of features extracted by local and global graph learning. **a** shows the t-SNE visualization of features extracted by iPiDA-A, iPiDA-G, and iPiDA-L, respectively. **b** and **c** show local context graphs for two example positive and two example negative piRNA-disease pairs in ${\mathbb{S}}_{\text{independent}}$, respectively. **d** shows the heatmap of features extracted by iPiDA-G and iPiDA-L for four example piRNA-disease pairs

The different features extracted by iPiDA-A, iPiDA-G, and iPiDA-L are further investigated and visualized in Fig. 3, revealing the following observations: (i) Compared to the spliced attribute features, the graph structural features can capture hidden association pattern between piRNAs and diseases and perform stronger discriminative ability. (ii) The pair features extracted by iPiDA-L are more discriminative and expressive compared to those by iPiDA-G. The global graph representation in iPiDA-G learns general features for each piRNA or disease node, which are then used to construct pair representations. Therefore, it is limited in its ability to differentiate pairs that share the same target piRNA or disease. In contrast, local graph representation in iPiDA-L captures pair features from their specific local-contextual graphs. It cannot only detect specific patterns across different piRNA-disease pair types, but also extract refined contextual semantics for target piRNA-disease pairs. For example, (piR-hsa-24486, Renal cell carcinoma) and (piR-hsa-24486, Parkinson disease) are two positive associations, showing distinct heatmap patterns compared to other two negative pairs. Furthermore, the local graphs constructed for the positive associations exhibit subtle differences, conforming to the discovery that piR-hsa-24486 shows differentially downregulated and upregulated expression in the two target diseases [32, 33], respectively.

### Performance comparison of various methods

Six cutting-edge methods include iPiDi-PUL [18], iPiDi-GCN [24], CLPiDA [28], iPiDi-SWGCN [27], ETGPDA [25], and PUTransGCN [26]. The web servers or source codes for these methods are readily available, facilitating an unbiased performance comparison. iPiDi-PUL identifies piRNA-disease associations using classical machine-learning algorithms with manual feature engineering process. The other five predictors adaptively learn pair features from the global piRNA-disease association graph using various graph neural networks.

**Fig. 4:**
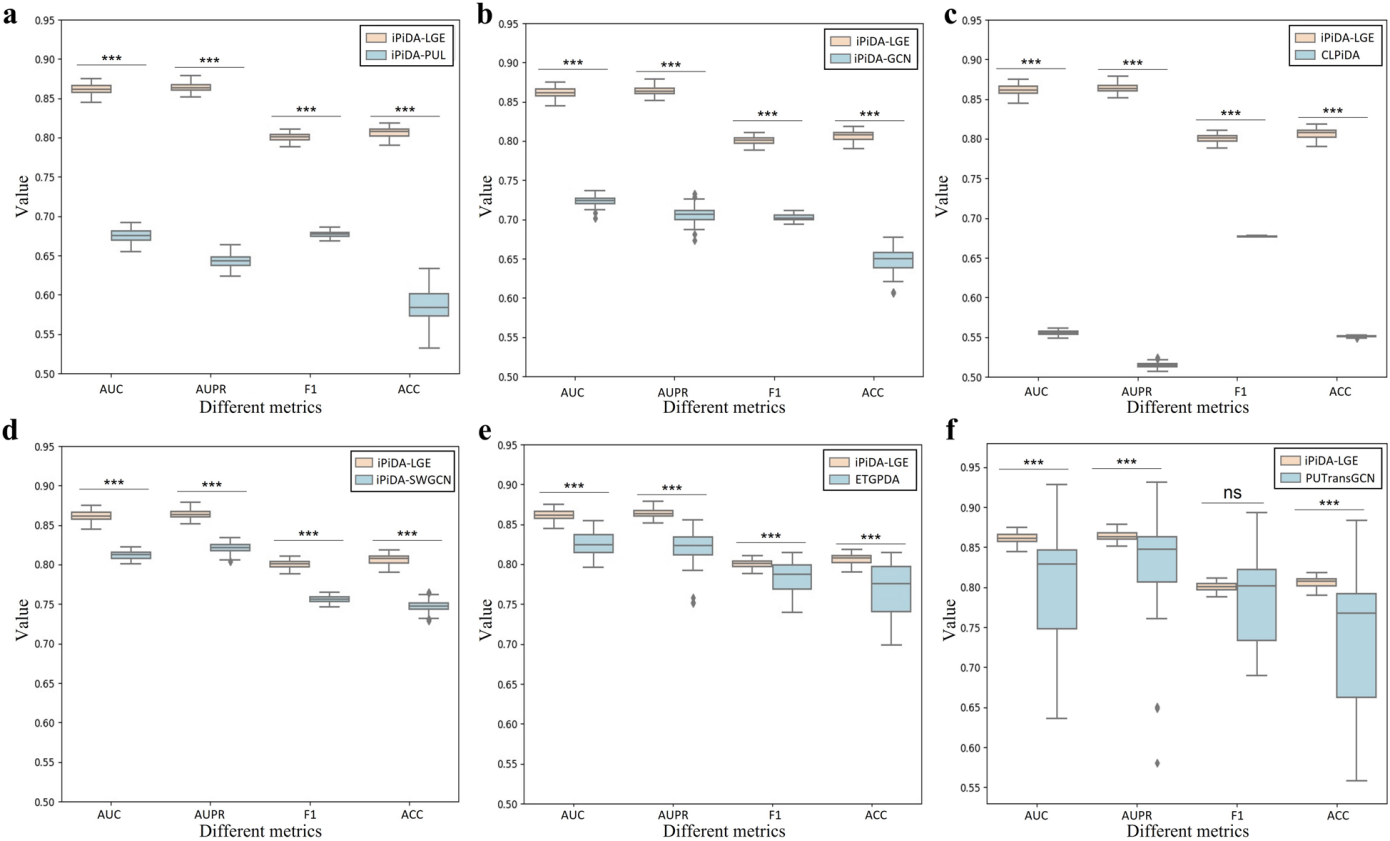
Comparison of different methods across four comprehensive metrics on 100 partitioned independent test sets. Wilcoxon rank-sum test is used to calculate the statistical difference between two groups of results. Comparisons with *p* values < 0.05 are marked with *, *p* values < 0.01 with **, *p* values < 0.001 with ***, and “ns” indicates no significant difference

To improve the reliability of method comparisons, we randomly partition the ${\mathbb{S}}_{\text{all}}^{+}$ 100 times according to the strategy described in section “Datasets,” creating 100 independent test sets. The prediction results obtained from different methods across these 100 independent test sets are then compared. The average comparison results are listed in Table 2, while the statistical test results are shown in Fig. 4. The findings indicate that the local and global graph ensemble learning framework used in iPiDA-LGE demonstrates significantly superior performance across most metrics.

**Table 2:** Performance comparison of different methods on 100 partitioned independent test sets

Methods	AUC	AUPR	F1	ACC	PRE	SPE	SEN
iPiDi-PUL	0.6749	0.6434	0.6771	0.5848	0.5556	0.2991	0.8705
iPiDA-GCN	0.7236	0.706	0.7023	0.6473	0.6086	0.4627	0.8319
CLPiDA	0.5555	0.5145	0.6771	0.5507	0.5285	0.1593	0.9422
iPiDA-SWGCN	0.8122	0.8211	0.7566	0.7476	0.7313	0.7106	0.7845
ETGPDA	0.8255	0.8232	0.7845	0.7707	0.7467	0.7100	0.8314
PUTransGCN	0.7990	0.8349	0.7821	0.7337	0.6818	0.5284	0.9389
iPiDA-LGE	0.8613	0.8637	0.8006	0.8066	0.8270	0.8370	0.7763

Leave-one-disease-out cross-validation is also conducted to evaluate the generalization ability of iPiDA-LGE. Different from focusing on uncovering missing associations within known diseases as previously discussed. In this scenario, all known associations with each target disease in ${\mathbb{S}}_{\text{all}}^{+}$ are firstly removed. The training set consists of known associations along with an equal number of unknown associations related to other 18 diseases. The trained model is then used to predict the positive and negative associations for each target disease. We compared the predicted results of different methods using leave-one-disease-out cross-validation. The average comparison results are shown in Additional file 1: Table S1, with the statistical test results shown in Additional file 1: Fig. S1. Compared to the scenario of identifying missing associations for known diseases, the performance of most methods in leave-one-disease-out cross-validation scenario is relatively unstable and shows decline, likely due to significant differences in the distribution of associations between different diseases and the incomplete network structure of the target disease. Nevertheless, iPiDA-LGE consistently outperforms other methods in comprehensive metrics such as AUC and AUPR and achieves superior or at least comparable performance in F1 score and accuracy.

### Case study

Case studies are implemented to further investigate the prediction quality of disease-associated piRNAs identified by iPiDA-LGE. We choose five significant diseases, specifically “Parkinson’s disease,” “cardiovascular disease,” “renal cell carcinoma,” “Alzheimer’s disease,” and “male infertility.” The top five predicted piRNAs associated with each target disease in the independent test dataset are provided in Table 3, indicating that all of them have biological literature supporting. The predicted disease-associated piRNAs show significantly differential expression between different sample groups. For instance, piR-hsa-28421 exhibits significantly elevated expression levels in cell samples derived from patients afflicted with Parkinson’s disease [32]. PiR-hsa-4946 shows abnormally downregulated in cardiovascular disease, and its expression in cardiomyocytes is more than 4 times higher than that in cardiomyocyte derived cells [34]. PiR-hsa-26731 and piR-hsa-20294 have been shown to exhibit differential expression levels in metastatic renal cell tumors compared to non-metastatic tumors, with piR-hsa-26731 being over fourfold lower and piR-hsa-20294 being over 40-fold higher [33]. In brain tissues of Alzheimer’s disease patients, piR-hsa-25783 exhibits an approximately eightfold increase in expression compared to that of normal individuals [35]. PiR-hsa-32046 is significantly downregulated in the seminal plasma of infertile patients against the fertile control groups [36]. In addition, multiple single nucleotide polymorphism (SNP) variations have been identified on most predicted disease-associated piRNAs, such as rs891709032, rs532580755, and rs998079688 have been identified on piR-hsa-4946 and rs149511548, rs898504240, rs938072950, and rs555921708 have been identified on piR-hsa-26731. These mutations may potentially trigger abnormal expression and affect their biological function [7].

**Table 3:** The top five piRNAs associated with different diseases predicted by iPiDA-LGE

Disease	Rank	piRNA^a^	Expression	Evidence^b^
Parkinson’s disease	1	piR-hsa-28421	Downregulated	29986767
	2	**piR-hsa-6841**	Downregulated	29986767
	3	piR-hsa-183	Downregulated	29986767
	4	piR-hsa-16796	Downregulated	29986767
	5	**piR-hsa-859**	Downregulated	29986767
Cardiovascular disease	1	**piR-hsa-4946**	Downregulated	27131603
	2	**piR-hsa-28185**	Downregulated	27131603
	3	piR-hsa-27715	Downregulated	27131603
	4	piR-hsa-2219	Downregulated	27131603
	5	**piR-hsa-27854**	Downregulated	27131603
Renal cell carcinoma	1	**piR-hsa-26731**	Downregulated	26071182
	2	**piR-hsa-27910**	Upregulated	26071182
	3	**piR-hsa-15980**	Upregulated	26071182
	4	**piR-hsa-31522**	Downregulated	26071182
	5	piR-hsa-20294	Upregulated	26071182
Alzheimer disease	1	**piR-hsa-25783**	Upregulated	28127595
	2	piR-hsa-27438	Upregulated	28654860
	3	**piR-hsa-27622**	Upregulated	28127595
	4	**piR-hsa-1100**	Upregulated	26934981
	5	**piR-hsa-16724**	Downregulated	28654860
Male infertility	1	**piR-hsa-32046**	Downregulated	27068805
	2	piR-hsa-7144	Downregulated	27068805
	3	**piR-hsa-7264**	Downregulated	27068805
	4	**piR-hsa-18608**	Downregulated	27068805
	5	**piR-hsa-18727**	Downregulated	27068805

^a^The piRNAs with multiple SNPs are highlighted in bold

^b^The PMIDs of supporting literature in PubMed have been provided

To evaluate the scalability of the proposed graph ensemble learning framework, it is applied to other two bio-entity association prediction tasks: miRNA-disease and circRNA-disease association prediction. For the miRNA-disease prediction task, HMDD v3.2 data [37] including 12,446 known associations between 853 miRNAs and 591 diseases is used to construct and evaluate iPiDA-LGE model. For the circRNA-disease prediction task, CircR2Disease v2.0 data [38] including 1820 experimentally validated associations covering 1429 circRNAs and 122 diseases is used to construct and evaluate proposed framework. In the both bio-entity association prediction tasks, we randomly extracted 20% of the known associations as test positive samples, while the remaining 80% were used as training positive samples. Then, we randomly selected an equal number of unknown pairs as negative samples for both the test and training sets. To ensure the reliability of the model performance evaluation, this dataset construction strategy was repeated 100 times, and the average performance metrics obtained by our proposed framework for both bio-entity association prediction tasks are listed in Additional file 1: Table S2. Specifically, the proposed graph ensemble learning framework achieved a high AUC of 0.9435 and AUPR of 0.9437 in the miRNA-disease association prediction task, and competitive performance with an AUC of 0.8623 and AUPR of 0.8621 in the circRNA-disease association prediction task. These results demonstrate the scalability of our proposed graph ensemble learning framework and its potential for further performance optimization in domain-specific tasks.

To facilitate researchers’ experimental validation and downstream analysis, we further investigated the top-ranked disease-associated miRNAs and circRNAs predicted by iPiDA-LGE. For the miRNA-disease prediction task, a graph ensemble predictor is constructed using all 12,446 known associations in HMDD v3.2 [37]. The trained predictor identified miRNAs associated with five significant diseases: “Alzheimer’s disease,” “breast neoplasms,” “colorectal neoplasms,” “leukemia,” and “liver neoplasms.” Additional file 1: Table S3 lists the top ten predicted miRNAs for each disease, along with their corresponding validation information. Notably, 42 of these predicted associations can be corroborated by HMDD v4.0 [39]. In contrast to miRNAs, circRNAs currently have fewer known associations with diseases. For the circRNA-disease association prediction task, ten important diseases are selected: “colorectal cancer,” “breast carcinoma,” “bladder carcinoma,” “lung adenocarcinoma,” “Alzheimer’s disease,” “esophageal squamous cell carcinoma,” “pancreatic cancer,” “glioblastoma,” “hepatoblastoma,” and “ovarian cancer.” During predictor construction, we implemented strict leave-one-disease-out cross-validation by excluding all known circRNA-disease associations for each target disease from the CircR2Disease v2.0 data. Additional file 1: Table S4 lists the top five predicted circRNAs for each target disease, along with relevant evidence. A total of 36 predictions were validated by CircR2Disease v2.0.

In general, iPiDA-LGE can predict novel piRNA-disease associations and provide candidate piRNAs for biological experiments to study pathological mechanisms. Moreover, the proposed local and global graph ensemble learning framework provides innovative computational insights and can be optimized for miRNA-disease and circRNA-disease association prediction tasks.

## Conclusions

Detecting piRNA-disease associations holds significant importance in understanding disease mechanisms and biomarker discovery. In this study, we propose a new computational method named iPiDA-LGE to detect piRNA-disease associations. In contrast to competing methods, it predominantly possesses the following advantages: (i) The global graph learning module incorporates side information like piRNA sequence and disease ontology and learns various basic predictors to construct supplementary heterogeneous association network. Therefore, the apparent sparsity issue of original association can be alleviated by enriching biological semantics. (ii) Diverging from general pair features obtained by global graph learning, the local graph learning module in iPiDA-LGE considers the specific functional mechanism of piRNAs in different diseases and encodes each target piRNA-disease pair as a local graph. As a result, it can learn a more discriminative summary representation by capturing specific contextual information. (iii) Lastly, iPiDA-LGE integrates the local and global graph representation learning, which can simultaneously achieve refined inferences based on local graphs and overarching judgments derived from global graphs, leading to an enhancement in predictive performance.

While iPiDA-LGE achieves superior capability in identifying reliable disease-related piRNAs, it is important to note that further validation through biological experiments is needed for some of the predicted associations that have not been confirmed. In addition, iPiDA-LGE may encounter challenges in elucidating comprehensive insights into how piRNAs influence disease development. Therefore, future enhancements will focus on providing more fine-grained guidance for understanding disease pathology at the piRNA level. Additional phenotypic and genotypic information, such as SNPs, expression profiles, piRNA targets, and multiple bio-entity associations, can be incorporated to construct a more comprehensive heterogeneous biological network. Moreover, the introduction of denoising techniques, causal inference, and interpretable mechanisms will improve the model robustness and biological implication.

## Methods

### Datasets

To construct a heterogeneous piRNA-disease graph, piRNA sequence and disease ontology information are downloaded from piRBase [40] and Disease Ontology [41] databases, respectively. In addition, the known associations between piRNAs and diseases are retrieved from MNDR v3.0 [42]. Following the removal of duplicate entries, a total of 11,981 experimentally validated associations involving 10,149 piRNAs and 19 diseases are compiled. The dataset can be denoted by:

1 $$\left\{\begin{array}{l}{\mathbb{S}}_{\text{all}}={\mathbb{S}}_{\text{all}}^{+}\bigcup {\mathbb{S}}_{\text{all}}^{-}\\ {\mathbb{S}}_{\text{all}}^{+}= {\mathbb{S}}_{\text{independent}}^{+}\cup {\mathbb{S}}_{\text{benchmark}}^{+}\\ {\mathbb{S}}_{\text{independent}}={\mathbb{S}}_{\text{independent}}^{+}\bigcup {\mathbb{S}}_{\text{independent}}^{-}\end{array}\right.$$

where ${\mathbb{S}}_{\text{all}}^{+}$ represents the positive set encompassing 10,149 known piRNA-disease associations, while ${\mathbb{S}}_{\text{all}}^{-}$ is the negative set composed of all possible unknown piRNA-disease pairs between 10,149 piRNAs and 19 diseases. Independent test set ${\mathbb{S}}_{\text{independent}}$ is constructed for testing different computational methods. Sequentially partition known associations related to each specific disease in ${\mathbb{S}}_{\text{all}}^{+}$ into five folds. One fold from all 19 diseases is integrated to form the independent positive set ${\mathbb{S}}_{\text{independent}}^{+}$, which is then combined with a randomly selected equal number of negative pairs from ${\mathbb{S}}_{\text{all}}^{-}$ to constitute the independent negative set ${\mathbb{S}}_{\text{independent}}^{-}$. The remaining four folds of positive associations, along with an equal number of negative pairs, are used to create ${\mathbb{S}}_{\text{benchmark}}$. To train predictor and optimize hyper-parameters, ${\mathbb{S}}_{\text{benchmark}}$ is further divided and can be denoted as:

2 $$\left\{\begin{array}{l}{\mathbb{S}}_{\text{benchmark}}= {\mathbb{S}}_{\text{train}}\cup {\mathbb{S}}_{\text{validation}}\\ {\mathbb{S}}_{\text{train}}={\mathbb{S}}_{\text{train}}^{+}\cup {\mathbb{S}}_{\text{train}}^{-}\\ {\mathbb{S}}_{\text{validation}}={\mathbb{S}}_{\text{validation}}^{+}\cup {\mathbb{S}}_{\text{validation}}^{-}\end{array}\right.$$

where the positive set in ${\mathbb{S}}_{\text{benchmark}}$ is divided into five folds, four folds constitute ${\mathbb{S}}_{\text{train}}^{+},$ and ${\mathbb{S}}_{\text{validation}}^{+}$ is composed of remaining portion. ${\mathbb{S}}_{\text{train}}^{-}$ and ${\mathbb{S}}_{\text{validation}}^{-}$ are comprised of randomly selected negative pairs, equating in number to the corresponding positive set. The datasets can be downloaded from http://bliulab.net/iPiDA-LGE/download/.

### Method overview

**Fig. 5:**
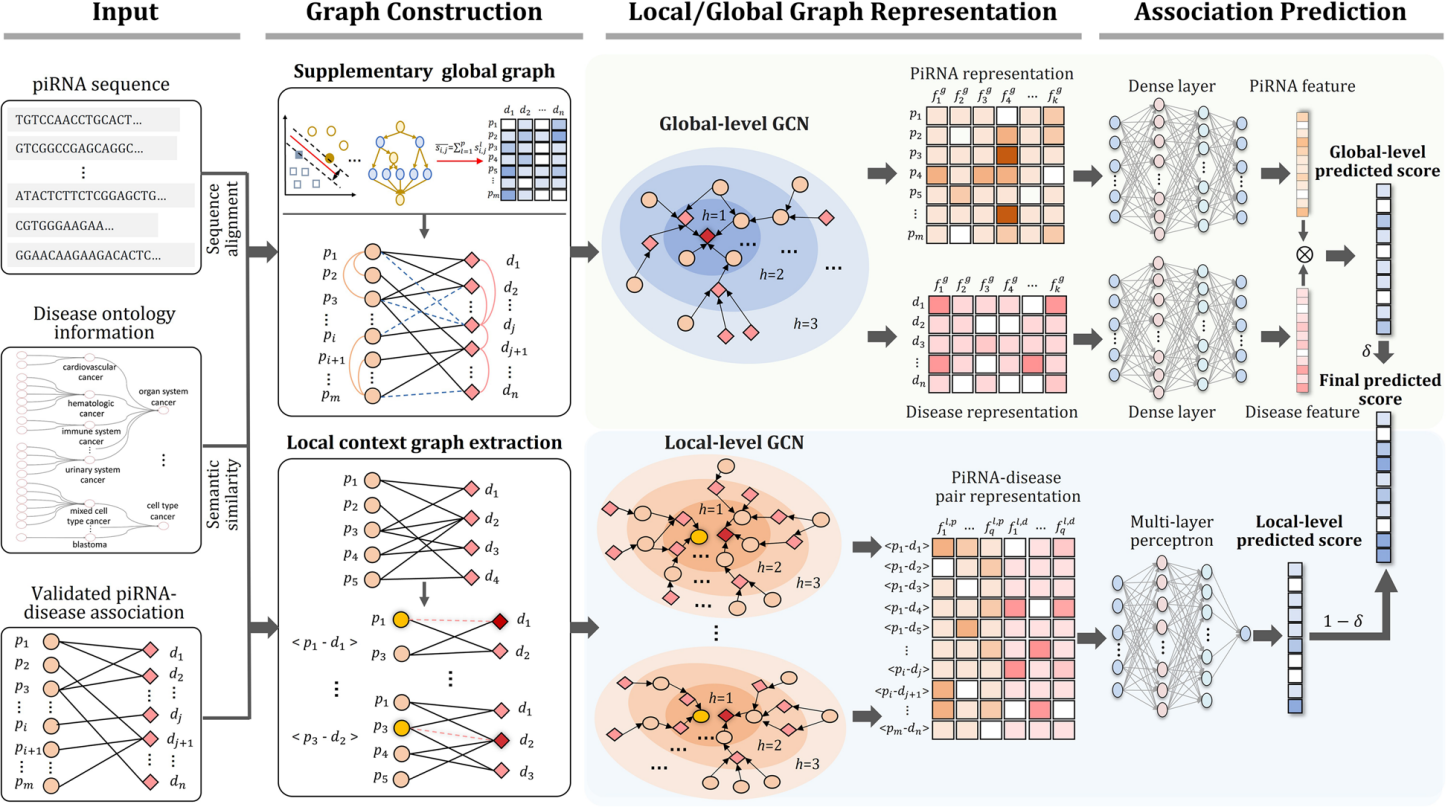
The framework of iPiDA-LGE. There exist three primary processes: (i) Graph construction. The global graph is constructed and supplemented with piRNA sequences, disease ontology knowledge, and validated piRNA-disease relationships. In addition, the local context graph for each target pair is extracted from original bipartite graph. (ii) Local/global graph representation. The representations of piRNA and disease nodes are captured by global-level GCN, while the pair representations are obtained by local-level GCN. (iii) Association prediction. The dense layer and multi-layer perceptron are applied to reduce feature dimensionality and calculate association scores. Finally, the global-level and local-level association scores are integrated with different weight coefficients to predict the relationships between piRNAs and diseases

As illustrated in Fig. 5, the iPiDA-LGE framework encompasses three primary steps: graph construction, graph representation, and association prediction. Detailed elaboration of each step will be provided in subsequent sections.

### Heterogeneous graph construction

To enhance the biological semantics of original piRNA-disease bipartite graph, the heterogeneous association graph is constructed and supplemented followed by our previous study [27]. Firstly, piRNA sequence similarity is obtained based on Smith-Waterman local alignment algorithm [43–45], and disease semantic similarity is calculated utilizing the DOSE package along with disease ontology knowledge [46]. Then, the global piRNA-disease association graph can be constructed by integrating three different types of edges: piRNA similarities, disease similarities, and piRNA-disease relationships. For alleviating limited neighbor information aggregation caused by high-sparse known association, the supplementarily weighted global graph is constructed. Fifteen predictors based on different machine-leaning algorithms and training samples are learned to score unknown piRNA-disease pairs, and the supplementary edges weighted with average scores predicted by fifteen basic predictors can be incorporated into original piRNA-disease association graph. The details of constructing a supplementary global piRNA-disease association graph can be referred to our previous study [27].

A local context graph for each target piRNA-disease pair is constructed for capturing local-specific structural features. Given the original piRNA-disease bipartite graph $$\text{G}$$, the local context graphs are constructed based on the enclosing subgraph extraction strategy [47]. Specifically, the piRNA and disease in a target pair ($$p,d)$$ are considered as core nodes, and their *h*-hop contextual neighbors are extracted by Breadth-First Search procedure. Then, the node-induced local graph $${\text{G}}_{p,d}^{h}$$ can be extracted from $$\text{G}$$ using corresponding core and context nodes. Particularly, if $${\text{G}}_{p,d}^{h}$$ contains target piRNA-disease association $$(p,d)$$, we should remove it to prevent information leakage. The main steps for extracting local context piRNA-disease graph are described in Algorithm 1.

**Figure:**
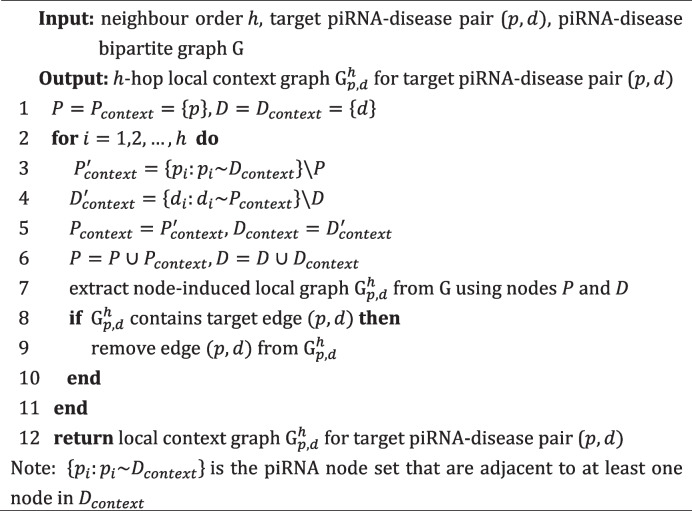
**Algorithm 1.** Local context graph extraction

The extracted local context piRNA-disease graphs are independent of global graph; a node labeling strategy is further applied to differentiate the node types and their hop stages [47]. The core nodes representing piRNAs are designated as 0, while those for diseases are labeled as 1. Other contextual nodes are assigned labels based on their node types and hop stages. A piRNA node at the *i*th hop can be labeled as 2*i*, while a disease node at the *i*th hop can be labeled as 2*i* + 1. Therefore, the nodes in local context graphs have distinguishable labels for following local-level graph representation learning.

### Global-level graph representation

Graph convolutional network (GCN) exhibits powerful capability to excavate intricate topological properties across diverse networks [48] and has been effectively applied to different bioinformatics tasks [49–52]. To capture global neighborhood features for piRNAs and diseases, GCN is adopted on the supplementary global association graph. At the *l*th layer, the node feature matrix ${\mathbf{H}}^{l}\in {\text{R}}^{(m+n)\times k}$ obtained by GCN can be represented as follows:

3 $${\mathbf{H}}^{l}=\sigma \left({\widetilde{\mathbf{D}}}^{-\frac{1}{2}}\widetilde{{\mathbf{A}}_{{\varvec{g}}}}{\widetilde{\mathbf{D}}}^{-\frac{1}{2}}{\mathbf{H}}^{l-1}{\mathbf{W}}^{l-1}\right)$$

and

4 $$\widetilde{{\mathbf{A}}_{{\varvec{g}}}}=\mathbf{I}+{\mathbf{A}}_{{\varvec{g}}}$$

5 $$\widetilde{\mathbf{D}}\left(i,i\right)=\sum_{j}\widetilde{{\mathbf{A}}_{{\varvec{g}}}}\left(i,j\right)$$

6 $${\mathbf{A}}_{{\varvec{g}}}=\left[\begin{array}{cc}{\mathbf{S}}_{\text{p}}& {\mathbf{A}}_{\text{s}}\\ {{\mathbf{A}}_{\text{s}}}^{\mathbf{T}}& {\mathbf{S}}_{\text{d}}\end{array}\right]$$

where ${\mathbf{A}}_{{\varvec{g}}}\in {\text{R}}^{(m+n)\times (m+n)}$ represents the piRNA-disease adjacency matrix for the global association graph, and $\mathbf{I}$ is the identity matrix. ${\mathbf{S}}_{\text{p}}\in {\text{R}}^{m\times m}$ and ${\mathbf{S}}_{\text{d}}\in {\text{R}}^{n\times n}$ denote matrices of piRNA and disease similarity, respectively, and ${\mathbf{A}}_{\text{s}}\in {\text{R}}^{m\times n}$ denotes the adjacency matrix for the supplementary piRNA-disease bipartite graph. $\sigma$ is defined as ReLU activation function. To acquire the attribute features of piRNA and disease nodes, we utilize the random walk with restart [53] algorithm on the matrices ${\mathbf{S}}_{\text{p}}$ and ${\mathbf{S}}_{\text{d}}$, and then dense layers are structured to uncover their hidden features. Thus, ${\mathbf{H}}^{l}$ is initialized by merging the attribute features of piRNAs and diseases, both with identical dimension. More details of global-level graph representation learning can be referred to our previous study [27].

### Local-level graph representation

Different from global-level graph representation, local-level graph representation learning aims to capture local contextual features for each piRNA-disease pair. Given a local context graph ${\mathbf{G}}_{p,d}$ for target piRNA-disease pair (*p*,*d*), each node feature vector ${\text{x}}_{i}^{l}$ learned at the *l*th layer can be calculated by [47]:

7 $$\text{x}_i^l=\sigma\left(\mathbf W_1^{l-1}\text{x}_i^{l-1}+{\sum_{j\in{\mathbb{N}}_i}\text{x}_j^{l-1}}\mathbf W_2^{l-1}\right)$$

where ${\mathbf{W}}_{1}^{l-1}$ and ${\mathbf{W}}_{2}^{l-1}$ are learnable parameter matrices, ${\mathbb{N}}_{i}$ represents the set of neighbors of node *i*, while $\sigma$ is defined as tanh activation function. For each node, its final representation ${\text{x}}_{i}$ can be obtained by concatenating *L* message passing layers:

8 $${\text{x}}_{i}=\text{concat}({\text{x}}_{i}^{1},{\text{x}}_{i}^{2},\dots ,{\text{x}}_{i}^{L})$$

Then, considering the greater importance of core nodes compared to other context nodes for the target piRNA-disease pair, the concatenate pooling layer is set to capture the local contextual features for ${\mathbf{G}}_{p,d}$:

9 $${\text{x}}_{p,d}=\text{concat}({\text{x}}_{p},{\text{x}}_{d})$$

where ${\text{x}}_{p}$ and ${\text{x}}_{d}$ are the final representations of target piRNA $p$ and disease $d$, respectively.

### PiRNA-disease association prediction

The global-level graph representation learning can capture general node features by considering the heterogeneous association and homogeneous similarity in the supplementary global graph. The local-level graph representation learning encodes each piRNA-disease pair as a sub-graph to aggregate refined contextual semantics. For comprehensively exploring association patterns between piRNAs and diseases, we integrate the local- and global-level graph representations to predict piRNA-disease association scores.

For the node features obtained from supplementary global piRNA-disease association graph, three dense layers with 400, 200, and 100 neurons are designed to extract high-level features. Then, for a target piRNA-disease pair (*p*,*d*), its association score can be predicted by inner product ${\mathbf{Y}}_{p,d}^{\text{global}}={\mathbf{h}}_{p}{\prime}{{\mathbf{h}}_{d}{\prime}}^{\text{T}}$, where ${\mathbf{h}}_{p}{\prime}$ and ${\mathbf{h}}_{d}{\prime}$ denote the features for target piRNA *p* and disease *d*. To avoid prediction bias problem, a variant loss function based on mean square error [54] is set:

10 $${\mathbf{L}}^{\text{global}}={\Vert {\mathbf{A}}^{{^{\prime}}}-{\mathbf{Y}}^{\text{global}}\Vert }_{\text{F}}^{2}+\mu {\Vert \mathbf{W}\Vert }_{2}^{2}$$

and

11 $${\mathbf{A}}_{p,d}^{{^{\prime}}}=\left\{\begin{array}{cc}0& \text{if }(p,d)\in {\mathbb{S}}_{\text{independent}}\\ 0& \text{if }{\mathbf{A}}_{p,d}=0\\ \alpha & \text{otherwise}\end{array}\right.$$

$\mathbf{A}\in {\text{R}}^{m\times n}$ denotes the adjacency matrix for the original piRNA-disease bipartite graph. ${\mathbf{A}}^{{^{\prime}}}$ and *μ* denote the variant adjacency matrix and decay factor, respectively. *μ* aims to regulate the regularization of trainable parameter matrix **W** in global-level graph representation learning.

For the piRNA-disease pair feature extracted from local context graph, the multilayer perceptron with 128 and 1 neurons is trained to predict piRNA-disease association scores:

12 $${\mathbf{Y}}^{\text{local}}={\mathbf{W}}_{2}\bullet \text{ReLU}\left({\mathbf{W}}_{1}\mathbf{X}+{b}_{1}\right)+{b}_{2}$$

where ${\mathbf{W}}_{1}$ and ${\mathbf{W}}_{2}$ are two learnable parameter matrices, while $\mathbf{X}$ denotes the piRNA-disease pair feature matrix. We employ the mean square error loss function to guide local context graph representation learning.

Given a test piRNA-disease pair (*p*,*d*), its final association score is computed by:

13 $${\mathbf{Y}}_{p,d}= {\delta \mathbf{Y}}_{p,d}^{\text{global}}+(1-\delta ) {\mathbf{Y}}_{p,d}^{\text{local}}$$

where $\delta$ is a parameter controlling the balance between global- and local-level predicted association scores.

### Performance evaluation

Different indicators, specifically the area under the receiver operating characteristics curve (AUC), area under the precision-recall curve (AUPR), F1 score, accuracy (ACC), precision (PRE), specificity (SPE), and sensitivity (SEN), are utilized to comprehensively estimate predictive performance [55–57]. AUC and AUPR respectively emphasize the trade-offs between sensitivity and specificity, and precision and recall across different threshold settings [58, 59].

## Supplementary Information

Additional file 1. Supplementary information. The performance comparison of different methods across metrics using leave-one-disease-out cross-validation is listed in Table S1. The comparison of different methods across four metrics using leave-one-disease-out cross-validation is shown in Fig. S1. The performance metrics obtained by iPiDA-LGE for other two bio-entity association prediction tasks is listed in Table S2. The top ten miRNAs associated with different diseases predicted by iPiDA-LGE is listed in Table S3. The top five circRNA associated with different diseases predicted by iPiDA-LGE is listed in Table S4.

## Data Availability

The iPiDA-LGE webserver is accessible at http://bliulab.net/iPiDA-LGE. The code and datasets used in this study can be found in online repositories. The name of the repository and accession number for the data reported in this paper is zenodo, 10.5281/zenodo.15080670 [60]. All data generated or analysed during this study are included in this published article, its supplementary information files, and publicly available repositories.

## Abbreviations

piRNA: PIWI-interacting RNA
GCN: Graph convolutional network
AUC: Area under the receiver operating characteristics curve
AUPR: Area under the precision-recall curve
ACC: Accuracy
PRE: Precision
SPE: Specificity
SEN: Sensitivity
