# Supplementary material for: iPiDA-LGE: a local and global graph ensemble learning framework for identifying piRNA-disease associations
Source: BMC Biol. 2025 May 9;23:119. doi: 10.1186/s12915-025-02221-y (PMC12065364; doi:10.1186/s12915-025-02221-y)
Supplement: Supplementary file 1 — Additional file 1. Supplementary information. The performance comparison of different methods across metrics using leave-one-disease-out cross-validation is listed in Table S1. The comparison of different methods across four metrics using leave-one-disease-out cross-validation is shown in Fig. S1. The performance metrics obtained by iPiDA-LGE for other two bio-entity association prediction tasks is listed in Table S2. The top ten miRNAs associated with different diseases predicted by iPiDA-LGE is listed in Table S3. The top five circRNA associated with different diseases predicted by iPiDA-LGE is listed in Table S4. [file 12915_2025_2221_MOESM1_ESM.docx]

**Additional File 1.**

The performance comparison of different methods across metrics using leave-one-disease-out cross-validation is listed in **Table S1**.

The comparison of different methods across four metrics using leave-one-disease-out cross-validation is shown in **Fig. S1**.

The performance metrics obtained by iPiDA-LGE for other two bio-entity association prediction tasks is listed in **Table S2**.

The top ten miRNAs associated with different diseases predicted by iPiDA-LGE is listed in **Table S3**.

The top five circRNA associated with different diseases predicted by iPiDA-LGE is listed in **Table S4**.

**Table S1**. Performance comparison of different methods across metrics using leave-one-disease-out cross-validation

| **Methods** | **AUC** | **AUPR** | **F1** | **ACC** | **PRE** | **SPE** | **SEN** |
| --- | --- | --- | --- | --- | --- | --- | --- |
| iPiDi-PUL | 0.6822 | 0.7194 | 0.6214 | 0.6023 | 0.6016 | 0.4955 | 0.6907 |
| iPiDA-GCN | 0.6392 | 0.6812 | 0.6605 | 0.5932 | 0.5658 | 0.3348 | **0.8330** |
| CLPiDA | 0.5888 | 0.7141 | 0.6951 | 0.6508 | 0.6502 | 0.4692 | 0.8138 |
| iPiDA-SWGCN | 0.7025 | 0.7400 | 0.6803 | 0.6418 | 0.6204 | 0.4581 | 0.8069 |
| ETGPDA | 0.7374 | 0.7910 | **0.7531** | **0.7404** | **0.7358** | **0.6628** | 0.8187 |
| PUTransGCN | 0.7025 | 0.7597 | 0.7331 | 0.7148 | 0.6983 | 0.6010 | 0.8190 |
| iPiDA-LGE | **0.8169** | **0.8471** | 0.7463 | 0.7373 | 0.7312 | 06545 | 0.8016 |


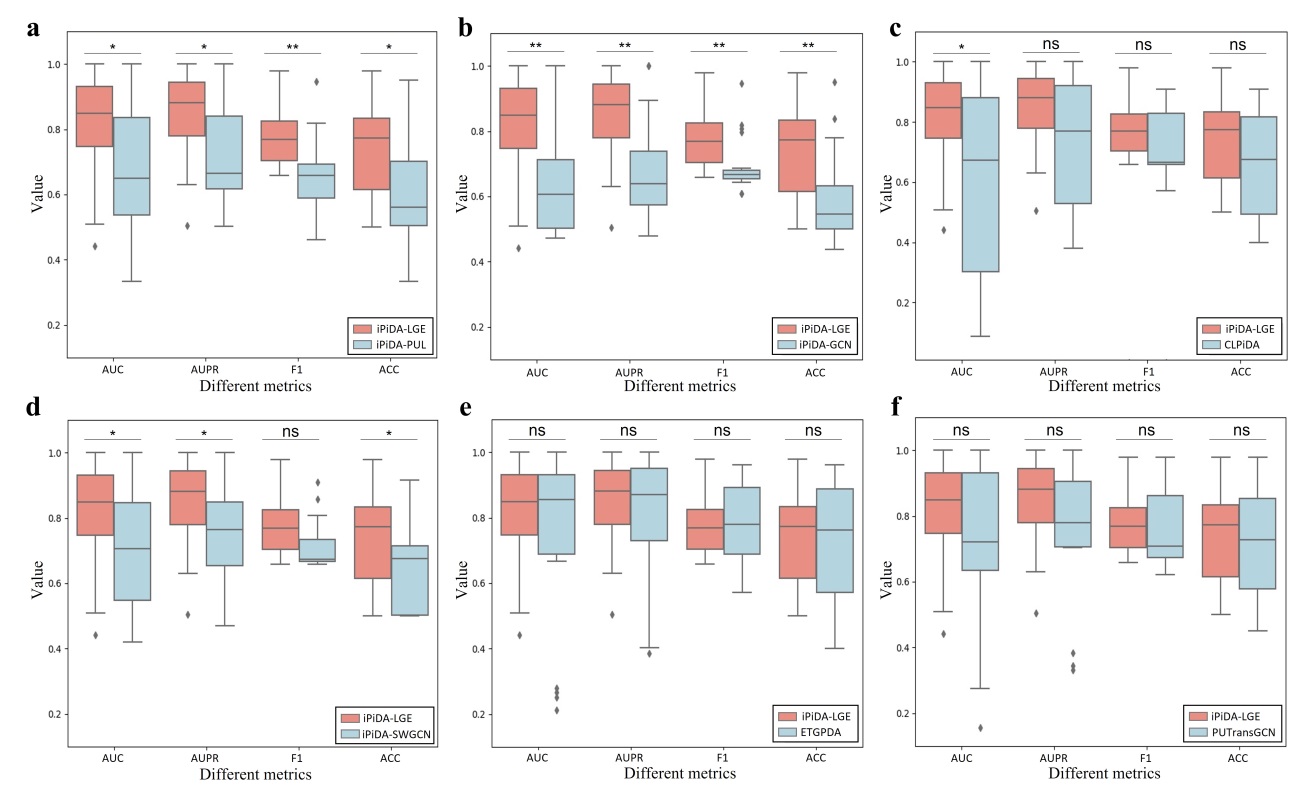


**Fig. S1. Comparison of different methods across four metrics using leave-one-disease-out cross-validation.** Wilcoxon rank-sum test is used to calculate the statistical difference between two groups of results. Comparisons with p-values < 0.05 are marked with *, p-values < 0.01 with **, p-values < 0.001 with ***, and 'ns' indicates no significant difference.

**Table S2**. Performance metrics obtained by iPiDA-LGE for other two bio-entity association prediction tasks

| **Association prediction tasks** | **AUC** | **AUPR** | **F1** | **ACC** | **PRE** | **SPE** | **SEN** |
| --- | --- | --- | --- | --- | --- | --- | --- |
| miRNA-disease | 0.9435 | 0.9437 | 0.8738 | 0.8701 | 0.8510 | 0.8424 | 0.8977 |
| circRNA-disease | 0.8623 | 0.8621 | 0.8027 | 0.7909 | 0.7673 | 0.7400 | 0.8417 |

**Table S3**. The top ten miRNAs associated with different diseases predicted by iPiDA-LGE

| **Disease** | **Rank** | **miRNA** | **Evidence code**^a^ | **Evidence**^b^ |
| --- | --- | --- | --- | --- |
| Alzheimer's disease | 1 | hsa-mir-150 | circulation_biomarker_diagnosis_up | 35811521 |
|  | 2 | hsa-mir-223 | lncRNA target | 32531989 |
|  | 3 | hsa-mir-126 | circulation_biomarker_diagnosis_ns | 33776746 |
|  | 4 | hsa-mir-221 | Unknown | Unconfirmed |
|  | 5 | hsa-mir-145 | Unknown | Unconfirmed |
|  | 6 | hsa-mir-22 | genetics_overexpression_suppress | 31713484 |
|  | 7 | hsa-mir-122 | circulation_biomarker_diagnosis_down | 36771341 |
|  | 8 | hsa-mir-146b | circulation_biomarker_diagnosis_ns | 32414413 |
|  | 9 | hsa-mir-210 | circulation_biomarker_diagnosis_up | 30734227 |
|  | 10 | hsa-mir-30a | Unknown | Unconfirmed |
| Breast neoplasms | 1 | hsa-mir-483 | epigenetics | 33446575 |
|  | 2 | hsa-mir-28 | genetics_knock down_promote | 34593318 |
|  | 3 | hsa-mir-300 | Unknown | Unconfirmed |
|  | 4 | hsa-mir-138-1 | Unknown | Unconfirmed |
|  | 5 | hsa-mir-422a | other | 30583076 |
|  | 6 | hsa-mir-186 | genetics_overexpression_suppress | 35251568 |
|  | 7 | hsa-mir-1297 | therapeutic target | 32319577 |
|  | 8 | hsa-mir-1271 | transcription factor target | 30823890 |
|  | 9 | hsa-mir-138-2 | Unknown | Unconfirmed |
|  | 10 | hsa-mir-154 | genetics_knock down_promote | 33023375 |
| Colorectal neoplasms | 1 | hsa-let-7d | genetics_GWAS | 32676506 |
|  | 2 | hsa-mir-98 | genetics_overexpression_promote | 30506722 |
|  | 3 | hsa-mir-296 | genetics_overexpression_promote | 34883316 |
|  | 4 | hsa-mir-30e | Unknown | Unconfirmed |
|  | 5 | hsa-mir-151a | target gene | 36366788 |
|  | 6 | hsa-let-7f-1 | genetics_knock down_suppress | 17965831 |
|  | 7 | hsa-mir-212 | genetics_overexpression_promote | 32697380 |
|  | 8 | hsa-mir-302b | other | 22384170 |
|  | 9 | hsa-mir-494 | other | 35008652 |
|  | 10 | hsa-mir-101-1 | genetics_knock down_promote | 22930392 |
| Leukemia | 1 | hsa-mir-222 | tissue_expression_ns | 31388255 |
|  | 2 | hsa-mir-106b | target gene | 19096009 |
|  | 3 | hsa-mir-146b | circulation_biomarker_diagnosis_down | 23286334 |
|  | 4 | hsa-mir-93 | genetics_knock down_promote | 34476495 |
|  | 5 | hsa-mir-106a | genetics_overexpression_promote | 32380791 |
|  | 6 | hsa-mir-132 | tissue_expression_up | 25645730 |
|  | 7 | hsa-mir-25 | genetics_knock down_promote | 36756106 |
|  | 8 | hsa-mir-29c | circulation_biomarker_diagnosis_down | 19144983 |
|  | 9 | hsa-mir-182 | genetics_knock down_promote | 36593968 |
|  | 10 | hsa-let-7g | lncRNA target | 34187521 |
| Liver neoplasms | 1 | hsa-mir-17 | genetics_GWAS | 32748943 |
|  | 2 | hsa-mir-20a | other | 33275223 |
|  | 3 | hsa-mir-145 | lncRNA target | 32440147 |
|  | 4 | hsa-mir-146b | therapeutic target | 19584283 |
|  | 5 | hsa-let-7d | Unknown | Unconfirmed |
|  | 6 | hsa-let-7g | other | 36154076 |
|  | 7 | hsa-mir-18a | genetics_overexpression_promote | 34221105 |
|  | 8 | hsa-mir-15a | genetics_overexpression_promote | 31099097 |
|  | 9 | hsa-mir-27a | circulation_biomarker_diagnosis_up | 36530488 |
|  | 10 | hsa-mir-206 | genetics_overexpression_promote | 32286127 |

^a^ The evidence codes in HMDD v4.0 have been provided.

^b^ The PMIDs of supporting literature in PubMed have been provided.

**Table S4.**The top five circRNA associated with different diseases predicted by iPiDA-LGE

| **Disease** | **Rank** | **circRNA** | **Expression** | **Evidence**^a^ |
| --- | --- | --- | --- | --- |
| Colorectal cancer | 1 | hsa_circ_0060927 | Up-regulated | 32803502 |
|  | 2 | hsa_circ_0000504 | Up-regulated | 28656150 |
|  | 3 | hsa_circ_0002138 | Down-regulated | 25624062 |
|  | 4 | hsa_circ_0053277 | Up-regulated | 31549406 |
|  | 5 | hsa_circ_0127801 | Up-regulated | 31496737 |
| Breast carcinoma | 1 | hsa_circ_0000646 | Up-regulated | 30810051 |
|  | 2 | hsa_circ_0000002 | Down-regulated | 30810051 |
|  | 3 | hsa_circ_0001006 | Up-regulated | 30810051 |
|  | 4 | hsa_circ_0025580 | Unknown | Unconfirmed |
|  | 5 | hsa_circ_0003159 | Unknown | Unconfirmed |
| Bladder carcinoma | 1 | hsa_circ_0020394 | Up-regulated | 30983072 |
|  | 2 | hsa_circ_0082582 | Down-regulated | 27484176 |
|  | 3 | hsa_circ_0025580 | Unknown | Unconfirmed |
|  | 4 | hsa_circ_0043603 | Unknown | Unconfirmed |
|  | 5 | hsa_circ_0005082 | Unknown | Unconfirmed |
| Lung adenocarcinoma | 1 | hsa_circ_0001320 | Down-regulated | 30787976 |
|  | 2 | hsa_circ_0000792 | Up-regulated | 29932500 |
|  | 3 | hsa_circ_0004062 | Down-regulated | 30787976 |
|  | 4 | hsa_circ_0025580 | Unknown | Unconfirmed |
|  | 5 | hsa_circ_0000264 | Up-regulated | 30787976 |
| Alzheimer's disease | 1 | hsa_circ_0007556 | Up-regulated | 33003364 |
|  | 2 | hsa_circ_0031258 | Down-regulated | 32315771 |
|  | 3 | hsa_circ_0003594 | Down-regulated | 30887246 |
|  | 4 | hsa_circ_0131235 | Up-regulated | 33704916 |
|  | 5 | hsa_circ_0003611 | Up-regulated | 32315771 |
| Esophageal squamous cell carcinoma | 1 | hsa_circ_0043603 | Down-regulated | 30674324 |
|  | 2 | hsa_circ_0025580 | Up-regulated | 31612046 |
|  | 3 | hsa_circ_0033988 | Unknown | Unconfirmed |
|  | 4 | hsa_circ_0055538 | Unknown | Unconfirmed |
|  | 5 | hsa_circ_0006948 | Up-regulated | 33630215 |
| Pancreatic cancer | 1 | hsa_circ_001587 | Up-regulated | 32878470 |
|  | 2 | hsa_circ_0006215 | Up-regulated | 29930719 |
|  | 3 | circ_0092314 | Up-regulated | 33842379 |
|  | 4 | hsa_circ_0071036 | Up-regulated | 33507122 |
|  | 5 | hsa_circ_0086375 | Down-regulated | 32366257 |
| Glioblastoma | 1 | hsa_circ_0043603 | Unknown | Unconfirmed |
|  | 2 | hsa_circ_0055538 | Unknown | Unconfirmed |
|  | 3 | hsa_circ_0033988 | Unknown | Unconfirmed |
|  | 4 | hsa_circ_0083682 | Down-regulated | 29967262 |
|  | 5 | hsa_circ_0077232 | Up-regulated | 29967262 |
| Hepatoblastoma | 1 | hsa_circ_0043603 | Unknown | Unconfirmed |
|  | 2 | hsa_circ_0055538 | Unknown | Unconfirmed |
|  | 3 | hsa_circ_0040081 | Down-regulated | 29414822 |
|  | 4 | hsa_circ_0017515 | Up-regulated | 29414822 |
|  | 5 | hsa_circ_0025580 | Unknown | Unconfirmed |
| Ovarian cancer | 1 | hsa_circ_0001095 | Down-regulated | 31623606 |
|  | 2 | hsa_circ_0000714 | Up-regulated | 33380810 |
|  | 3 | hsa_circ_0002711 | Up-regulated | 31589961 |
|  | 4 | hsa_circ_0051240 | Up-regulated | 30945557 |
|  | 5 | hsa_circ_0001756 | Up-regulated | 31589961 |

^a^ The PMIDs of supporting literature in PubMed have been provided.
